# Distribution of Muscle Mass and Fat Mass to Identify the Risk of Sarcopenia and Sarcopenic Obesity in Adults via Machine Learning

**DOI:** 10.3390/healthcare14121746

**Published:** 2026-06-17

**Authors:** Rodrigo Yáñez-Sepúlveda, Frano Giakoni-Ramírez, Juan Pablo Alarcón-Cortés, Juan David Paucar-Uribe, Boryi A. Becerra-Patiño, Eduardo Guzmán-Muñoz, Nicole Aguilera-Martínez, Vicente Javier Clemente-Suárez, José Francisco López-Gil

**Affiliations:** 1 Faculty Education and Humanities, Universidad Andres Bello, Viña del Mar 2520000, Chile; rodrigo.yanez.s@unab.cl (R.Y.-S.); frano.giakoni@unab.cl (F.G.-R.); 2 Department of Sport Sciences, Faculty of Sport and Health Sciences, Fit Generation Research Institute, AD500 Andorra la Vella, Andorra; 3 Departamento de Rehabilitación, Intervención y Abordaje Terapéutico, Universidad de Playa Ancha, Valparaíso 2340000, Chile; juan.alarcon@upla.cl; 4 Faculty of Physical Education, National Pedagogical University, Bogotá 480100, Colombia; jdpaucaru@upn.edu.co (J.D.P.-U.); babecerrap@pedagogica.edu.co (B.A.B.-P.); 5 Programa de Doctorado en Ciencias de la Actividad Física y del Deporte, University of Murcia, 30720 Murcia, Spain; 6 Escuela de Kinesiología, Facultad de Salud, Universidad Santo Tomás, Talca 3460000, Chile; eguzmanm@santotomas.cl; 7 Escuela de Pedagogía en Educación Física, Facultad de Educación, Universidad Autónoma de Chile, Talca 3460000, Chile; 8 Escuela de Nutrición y Dietética, Facultad Ciencias de la Salud, Universidad Católica del Maule, Talca 3460000, Chile; naguilera@ucm.cl; 9 Grupo de Investigación en Cultura, Educación y Sociedad, Universidad de la Costa, Barranquilla 080002, Colombia; vctxente@yahoo.es; 10 School of Medicine, Universidad Espíritu Santo, Samborondón 092301, Ecuador; 11 Vicerrectoría de Investigación y Postgrado, Universidad de Los Lagos, Osorno 5300000, Chile

**Keywords:** body composition, sarcopenia, sarcopenic obesity, fat-to-muscle ratio, appendicular skeletal muscle mass index, unsupervised machine learning

## Abstract

**Objective**: This study aimed to evaluate a sample of adults to define phenotypes on the basis of the association between adiposity and muscle mass via an unsupervised machine learning model. **Methods**: A cross-sectional study was conducted with 2710 adults, comprising women (*n* = 1907) and men (*n* = 803). The variables analyzed were absolute and relative muscle mass, the muscle mass index, appendicular skeletal muscle mass, body fat percentage, total fat mass, the trunk-to-limb fat ratio, and the fat-to-muscle ratio. K-means clustering was performed by sex. The clusters were validated via inertia and silhouette measures, and comparisons were performed via post hoc tests based on variance and Tukey’s test. **Results**: Different phenotypes were identified by sex. In women, a phenotype characterized by compensatory muscle, normal/lean, and sarcopenic obesity is observed, whereas in men, phenotypes characterized by metabolic obesity, athletic/protector, and sarcopenia risk/lean are identified. For women, the sarcopenic obesity phenotype is characterized by the highest adiposity burden and a higher fat-to-muscle ratio (FMR), indicating a disproportion between fat mass and muscle quality. In men, the sarcopenia risk/lean phenotype is defined as having limited muscle mass and an unfavorable FMR, despite having body mass index (BMI) values comparable to those of nonobese individuals. Across the whole sample, women presented higher FMR values and greater variability than men did. All between-phenotype differences were statistically significant (*p* < 0.001). **Conclusions**: Unsupervised machine learning identified biologically distinct body composition phenotypes that were not adequately captured by BMI alone.

## 1. Introduction

Metabolic health and health-related physical fitness during adulthood are determined by a wide range of biological and behavioral factors, among which body composition (BC) is a central component across the lifespan [1,2]. In addition to being a static characteristic, BC reflects the dynamic balance between skeletal muscle and adipose tissue, which is strongly influenced by aging, physical inactivity, and prolonged sedentary behavior. Together with a disproportionate accumulation of adipose tissue, evidence indicates that these factors promote an accelerated decline in skeletal muscle mass, thereby progressively shifting the organism toward a less favorable metabolic profile [3,4]. The loss of muscle mass and excessive gain of body fat led to metabolic homeostasis disruption, reduced functional capacity, and an increased risk of chronic diseases, including cardiovascular conditions, frailty, disability, and insulin resistance [5,6,7].

Despite the complexities arising from the relationships between different tissue processes, body mass index (BMI) continues to be used as a standardized measure for nutritional and metabolic analysis across various population groups; however, BMI is based on estimates of body size without distinguishing between fat mass and lean mass or their relative distribution in each individual [8,9,10]. This approach results in substantial differences in the determination of BC profiles grouped under similar BMI, without considering metabolic and functional risks. It is important to analyze this difficulty, specifically with respect to sarcopenic obesity, as it is characterized by minimal muscle mass and high adiposity, which may not be adequately assessed by conventional anthropometric indices alone [11]. In this context, relying exclusively on BMI could hinder the identification of high-risk phenotypes.

Consequently, there is a need to improve BC assessment to identify interactions among different tissues and, thereby, facilitate the identification of indicators that provide more information on adiposity and relative muscle quality, as well as the functional load that the musculoskeletal system can sustain. Various indices can provide more information, notably, the appendicular skeletal muscle mass index (ASMI), relative skeletal muscle mass (RSM), and fat-to-muscle ratio (FMR), all of which provide much more clinically relevant information than BMI alone [12,13]. These indices provide a much more comprehensive perspective on the evaluation of the balance between contractile tissue and adiposity, particularly because the interaction between these components does not follow a linear pattern [14]. Otherwise, since both contractile and adipose tissues generate specific physiological interactions, traditional indices are less sensitive for distinguishing differences across various phenotypes. To this end, new methodological approaches are needed to define different phenotypes based on indices such as the ASMI, RSM, and FMR.

In this context, models based on artificial intelligence (AI) and unsupervised machine learning (ML) have emerged as tools capable of identifying patterns through data categorization. Among these, the K-means clustering algorithm has been used as a methodological approach characterized by the exploration of hidden data within massive datasets to generate a stratification process since phenotypes derived from BC analysis [15,16]. This type of analysis aims to overcome predefined cutoff points or the evaluation of isolated variables. Thus, the unsupervised model seeks to identify the interaction between muscle mass and fat mass through data analysis via an integrative approach. The aim is to analyze the presence of transient or subclinical phenotypes that may be at risk of metabolic or functional decline.

Although sarcopenia has been extensively studied in older populations and risk stratification frameworks have been developed primarily for this age group [17,18], considerably less attention has been given to the identification of adverse muscle–fat phenotypes in young and middle-aged adults. This represents an important gap in literature, since early adulthood and midlife may constitute critical periods during which unfavorable BC trajectories begin to consolidate. Detecting such phenotypes before the onset of more advanced deterioration could improve risk profiling and support the implementation of timelier and more targeted preventive strategies. Therefore, the aim of the present study was to stratify the adult population into specific phenotypes based on the interaction between muscle mass and adiposity. It was hypothesized that unsupervised ML could identify distinct BC phenotypes in adults based on the interaction between muscle mass and adiposity, some of which may reflect less favorable profiles associated with sarcopenia-related risk.

## 2. Materials and Methods

### 2.1. Study Design and Participants

This cross-sectional study [19] applied unsupervised ML to identify BC phenotypes associated with sarcopenic obesity risk based on the quality and distribution of muscle and adipose tissue. The study sample comprised 2710 adults, including 1907 women (70.4%) and 803 men (29.6%), with a mean age of 37.72 years.

All analyses were conducted using Python 3.10.11 (Python Software Foundation, Wilmington, DE, USA) [20]. Widely used scientific libraries were employed throughout the analytical workflow, including Pandas (version 1.5.3) and NumPy (version 1.23.5) for data handling, Scikit-learn (version 1.2.2) for clustering procedures and model validation, SciPy (version 1.10.1) for statistical testing, and Matplotlib (version 3.7.1) together with Seaborn (version 0.12.2) for density plots and high-resolution radar visualizations.

### 2.2. Variables

The methodological approach used transformed the primary BC variables into various biomarkers that were standardized on the basis of allometric and functional criteria. Thus, the resulting variables were the ASMI, SMI, FMR, RSM, and the ratio derived from the analysis of the trunk and limbs. The ASMI variable was used to minimize the bias introduced by height in the assessment of appendicular muscle reserve and was calculated as the appendicular skeletal muscle mass (kg) divided by height squared (m^2^), following established allometric conventions. The FMR was calculated as the ratio of total fat mass to skeletal muscle mass and was used as an indicator of metabolic quality by quantifying the lipid load per unit of contractile tissue. RSM was calculated as the ratio of skeletal muscle mass to total body weight, expressed as a percentage. The SMI (skeletal muscle index) was calculated as total skeletal muscle mass (kg) divided by height squared (m^2^). Finally, the relationship between trunk fat mass and limb fat mass was analyzed by the ratio of trunk fat mass to limb fat mass. It is used as an indicator of centralized adipose mass and the potential risk of visceral inflammation.

The entire dataset was preprocessed before clustering was performed. Thus, variables measured on different scales, such as body mass in kilograms and body fat percentage, were standardized via Z scores to ensure that each characteristic would contribute equally to the metric analyzed by the clustering algorithm. This standardization process was performed via the following formula:

z = (x − μ)/σ

where μ represents the sample mean and σ represents the standard deviation. This transformation process shifts the variables toward zero and scales them to a unified variance, allowing the algorithm to assess the variation between muscle mass and body fat on a comparable basis. Moreover, missing values were analyzed via imputation procedures prior to clustering to subsequently consolidate the biomarkers derived from the entire multivariate dataset required for clustering. K-means clustering was used as an unsupervised learning method, initially employing k-means++ to improve centroid selection and minimize the probability of convergence toward local minima. The hyperparameters were defined as follows: three clusters by sex (K = 3), a maximum of 500 iterations per run, and 20 random initializations (n_init = 20) to ensure stability across different runs. The Euclidean distance was used in the multivariate feature space. Similarly, the appropriate number of clusters was defined based on two additional criteria: (i) the elbow method (inertia and the point of diminishing returns) and (ii) the silhouette coefficient to analyze cohesion and the union and disunion of the different clusters. Higher silhouette values were associated with more consistent cluster groupings. The silhouette scores obtained (0.317 for men; 0.307 for women) are consistent with expectations for biological phenotyping studies, where body composition variables exist on a continuum rather than as hyper-separated categories. Values in this range (0.20–0.50) indicate partial cluster overlap at biological margins, which is physiologically expected given the gradual transition between phenotypic states. Bootstrap stability analysis confirmed reproducibility of the cluster structure across 1000 replications, with narrow confidence intervals (men: [0.301, 0.341]; women: [0.297, 0.320]), supporting the robustness of the identified phenotypes (Table A2).

### 2.3. Statistical Analysis

To optimize the sensitivity and reproducibility of the different identified phenotypes, a validation and stability process was employed. This process involved performing bootstrap resampling to analyze the consistency of the clusters and training the model specifically for each sex to account for sexual dimorphism and ensure physiological consistency between women and men. The subsequent statistical analyses included one-way analysis of variance (ANOVA) to determine whether there were statistically significant differences in the mean values of the various variables among the three phenotypes. A post hoc test was subsequently performed via pairwise comparisons with Tukey’s honestly significant difference (HSD) test. Letters were assigned to indicate significance (a, b, c) to control Type I error. Statistical significance was set at α = 0.05. Each of the cluster validation metrics is presented in Table 1.

## 3. Results

The initial characterization of the total sample is presented in Table 2. The table shows that there are statistically significant differences in the anthropometric and BC variables with respect to sexual dimorphism for all the variables (*p* < 0.001). Women are characterized by a greater mean age (38.14 ± 11.73 years) and a metabolic profile represented by greater relative adiposity (39.84% ± 7.53) and a higher FMR (1.25 ± 0.38). This represents a comparatively greater lipid load per unit of muscle tissue than that in men. Moreover, men presented greater musculoskeletal mass, as evidenced by higher ASMI values (8.65 ± 0.69 kg/m^2^) and greater absolute muscle mass (36.36 ± 4.34 kg). Men, in turn, presented a lower FMR (0.59 ± 0.31), which may indicate greater metabolic efficiency despite greater accumulation of central fat.

Cluster analysis of the men cohort, summarized in Table 3, identified three biologically distinct BC phenotypes (*p* < 0.001), revealing a clinically relevant gradient of metabolic and musculoskeletal risk. The Athletic/Protector phenotype (*n* = 450) presented the most favorable profile, characterized by the highest relative muscle mass and the lowest FMR, suggesting a robust metabolic reserve and preserved structural integrity. In marked contrast, the metabolic obesity phenotype (*n* = 181) showed a pronounced homeostatic imbalance. Despite relatively high absolute muscle mass, these individuals exhibited substantial adiposity, nearly tripling the fat burden observed in the athletic group and reaching a clearly pathological BMI (35.37 ± 4.48 kg/m^2^). Finally, the sarcopenia risk/lean phenotype (*n* = 172) represented the youngest subgroup (33.73 ± 14.12 years) and appeared to reflect a more subtle but potentially clinically relevant vulnerability. Although BMI remained relatively moderate (24.54 ± 3.12 kg/m^2^), this group presented the lowest absolute muscle mass in the male cohort (31.84 ± 3.24 kg) together with a relatively low FMR (0.55 ± 0.25), which is consistent with an ‘invisible’ sarcopenia-related profile that may predispose individuals to early frailty and metabolic deterioration.

In the female cohort (*n* = 1907), the clustering algorithm identified three phenotypes with markedly heterogeneous risk profiles (*p* < 0.001 for all variables), underscoring the relevance of muscle quality beyond total body weight (Table 4). The sarcopenic obesity phenotype (*n* = 650) emerged as the group with the most adverse metabolic profile, characterized by a severe disproportion between adipose and muscle tissue. Although the absolute muscle mass was broadly comparable to that of the other groups (26.13 kg), this tissue was exposed to a critical adipose burden of 43.63 kg. Consequently, this phenotype exhibited a pathological FMR of 1.67 ± 0.23, suggesting substantial impairment in muscle quality associated with chronic fat overload. In contrast, the normal/lean group (*n* = 684), which was the youngest subgroup in the cohort (36.04 years), represented the most favorable reference profile, with the lowest body weight (62.93 kg) and BMI (25.24 kg/m^2^), together with superior overall metabolic efficiency. Finally, the compensatory muscle phenotype (*n* = 573) revealed a distinct physiological adaptation, comprising women with the greatest mean stature (1.61 m) and elevated relative muscle mass (36.41%). However, this group also had an overweight BMI (28.05 kg/m^2^) and an increased trunk-to-limb fat ratio (1.28 ± 0.07), suggesting a more centralized fat distribution that may warrant metabolic monitoring despite its apparently more robust muscular profile.

**Figure 1:**
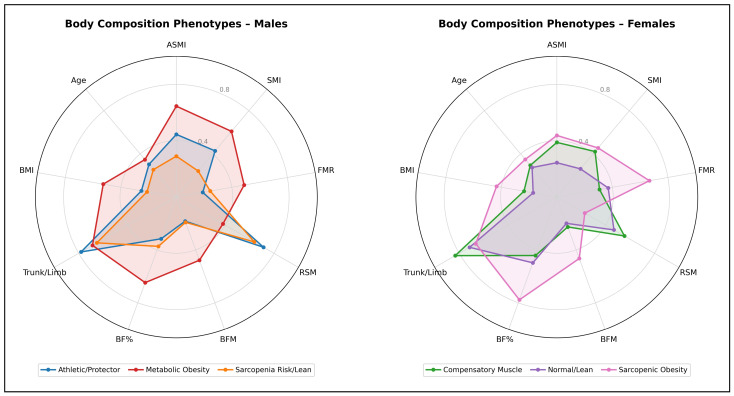
Sarcopenia-related body composition phenotypes in males and females. ASMI: Appendicular Skeletal Muscle Mass Index; BF%: Body Fat Percentage; BFM: Body Fat Mass; BMI: Body Mass Index; FMR: Fat-to-Muscle Ratio; RSM: Relative Skeletal Muscle mass; SMI: Skeletal Muscle Index; Trunk/Limb: Trunk-to-Limb Fat Ratio.

Radar charts function as metabolic and structural fingerprints by enabling the simultaneous visualization of the complex interactions among the nine key variables defining each phenotype. The phenotype analysis for men (Figure 1) revealed a contrast between the athletic and protective profiles, which extends across the various axes related to muscle density. The metabolic obesity profile shifts toward the dimensions with the highest scores for obesity and FMR. Moreover, the sarcopenia/lean mass risk profile exhibited a more reduced pattern associated with greater overall vulnerability. In women, the radar chart reveals the disproportion of the sarcopenic obesity phenotype, which is characterized by variables of fat mass, age, and metabolic load, whereas a pattern of muscular efficiency is not clearly observed. This representation confirms that the phenotypes identified by the model used in the present study vary not only in relation to isolated values but also in reference to the different biological configurations resulting from increased fat at the expense of muscle quality and proportionality.

**Figure 2:**
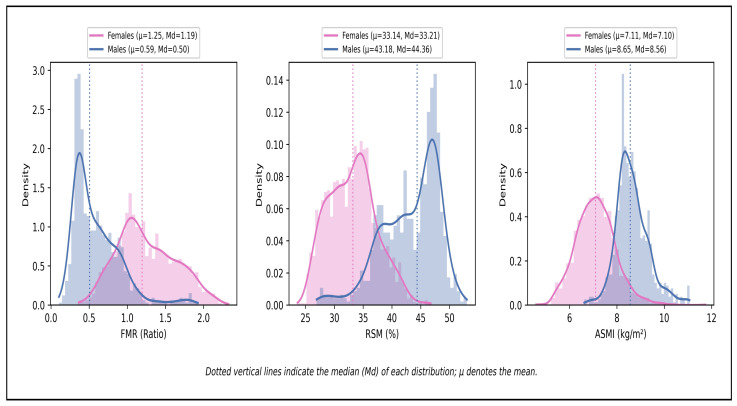
Sex-specific kernel density distribution of the fat-to-muscle ratio (FMR). Blue curve: men (n = 803); pink curve: women (n = 1907).

Figure 2 shows the sex-specific distribution of FMR via density curves. The blue curve represents men, whereas the pink curve represents women. The height of each point on the curve reflects the relative frequency of that value within each group. Men presented lower FMR values, which were mainly concentrated between approximately 0.3 and 0.7, with a peak at approximately 0.45–0.50. In contrast, women presented higher FMR values, predominantly between 0.8 and 1.5, with a peak at approximately 1.0–1.1. These findings indicate that women have a higher fat–muscle ratio than men do, while the broader female distribution also suggests greater variability in the FMR (Figure 2).

**Figure 3:**
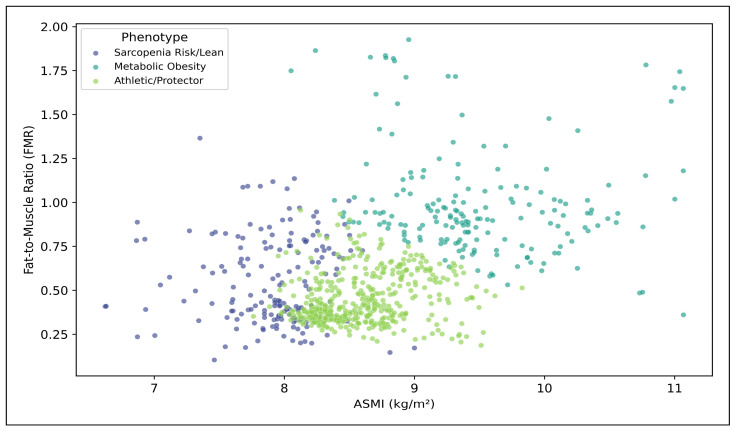
Body composition (BC) phenotypes in men, as identified by K-means clustering.

**Figure 4:**
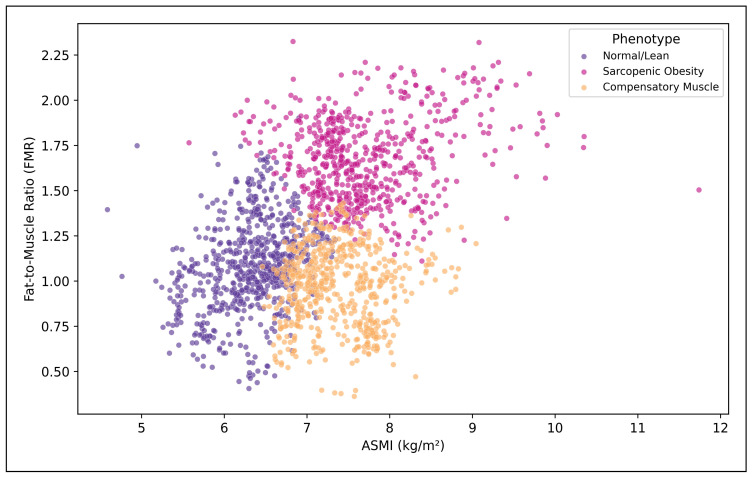
Body composition (BC) phenotypes in women, as identified by K-means clustering.

A weak-to-moderate positive association was observed between relative skeletal muscle mass and central fat distribution in both sexes. Men presented a greater average muscle mass together with a wider range of trunk fat distributions, whereas women presented a lower relative muscle mass and a clearer separation between BC categories. In this context, sarcopenic obesity is characterized by reduced muscle mass in combination with specific fat distribution patterns (Figure 3 and Figure 4).

**Figure 5:**
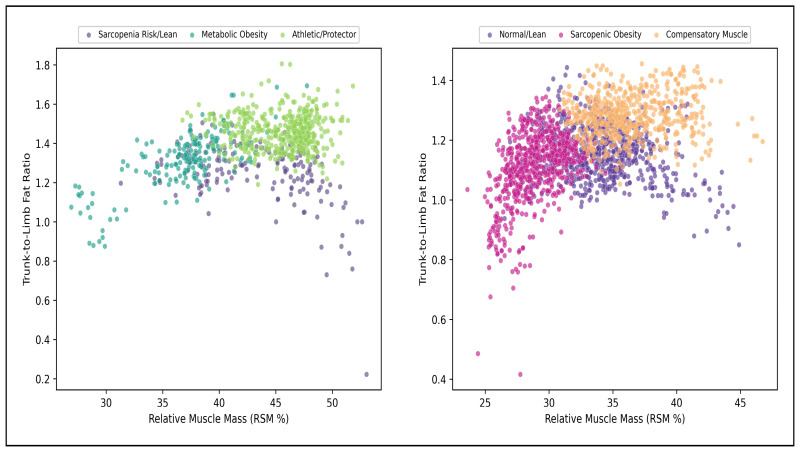
Distribution of body composition categories based on fat mass and muscle mass across the full sample (n = 2710), stratified by sex.

Figure 5 illustrates the distribution of different body types based on an analysis of muscle mass and fat distribution. This analysis shows that neither variable alone explains BC, indicating that the optimal condition is “Normal/Lean,” with “Intermediate” serving as a compensatory category, and the highest risk being associated with sarcopenic obesity.

**Figure 6:**
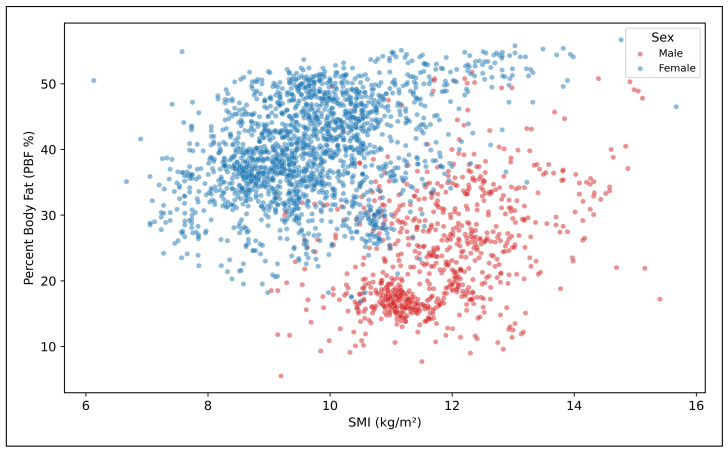
Skeletal muscle index (SMI, kg/m^2^) vs. percent body fat in men and women (n = 2710).

A clear sex-related difference was observed, with men showing higher SMI values and lower body fat percentages, whereas women displayed the opposite pattern. An intermediate range was identified (SMI ≈ 9–12) in which men and women may partially overlap. Nevertheless, the female distribution clearly shifted toward higher body fat values and lower muscle index values (Figure 6).

## 4. Discussion

The present study stratified a sample of 2710 adults into BC phenotypes via an unsupervised ML approach based on K-means clustering. The main objective was to identify adult phenotypes defined by the interaction between muscle mass and adiposity that could reveal early risk profiles beyond conventional anthropometric classification. In line with this objective, the findings support the study hypothesis by showing that the combined use of the ASMI and FMR enabled the identification of distinct phenotypes with potentially unfavorable metabolic and musculoskeletal profiles that would have remained partially concealed under traditional BMI-based assessment. In particular, the male sarcopenia risk/lean phenotype presented a markedly compromised muscular profile despite maintaining a BMI within a range that might not immediately raise clinical concern, whereas the female sarcopenic obesity phenotype presented a markedly elevated FMR, suggesting substantial impairment in muscle quality despite preserved absolute muscle mass. 

Analyses based on the interaction between the ASMI and FMR indicators using AI models, specifically unsupervised ML models, could serve as a more sensitive strategy for assessing the risks of sarcopenia and myosteatosis in the adult population. The findings of this study are consistent with those of various studies that have concluded that the validity of BMI as an independent indicator of metabolic health and BC has limitations regarding adequately assessing the distribution of various phenotypes [21]. The specific novelty of this study lies not in proposing entirely new phenotypic categories, but in the data-driven, multi-index quantification of the muscle–fat imbalance within each phenotype using the FMR as a continuous metabolic quality indicator, rather than as a binary classifier. 

The ‘sarcopenia risk/lean’ male phenotype, for instance, presents BMI values within the nonobese range yet demonstrates objectively compromised muscle reserve and an elevated lipid-to-contractile tissue ratio—a clinically “invisible” profile that no single conventional threshold would detect. Furthermore, the identified phenotypes emerge from the covariance among multiple continuous indices (ASMI, FMR, RSM, trunk-to-limb fat ratio), providing a multidimensional perspective that standard single-variable classifications cannot replicate. These phenotypic profiles should be interpreted as risk profiles for future surveillance rather than definitive clinical diagnoses, and prospective studies linking these clusters to functional outcomes, metabolic biomarkers, and longitudinal trajectories are warranted.

Previous studies have shown that BMI may misclassify individuals with high muscularity as overweight or obese while failing to detect individuals with low muscle reserve or concealed sarcopenia [22,23]. However, unlike earlier approaches based primarily on fixed cutoff points, the present study applied a dynamic clustering model capable of identifying nonlinear and multidimensional patterns in BC data. Although sarcopenia has traditionally been conceptualized as a condition that primarily affects older adults [17], the current results suggest that deterioration in muscle quality, reflected here by an unfavorable FMR, may already be detectable in mid-adulthood. This interpretation aligns with emerging evidence on earlier-onset sarcopenia-related trajectories associated with obesity, low physical activity, and other determinants of impaired muscle health in individuals aged approximately 40–59 years [24].

This study highlights high-risk phenotypes, particularly sarcopenic obesity in women and metabolic obesity in men. This could be associated with various physiological processes, including lipid toxicity, tissue homeostasis, and anabolic resistance [25,26,27]. Although the available human literature remains limited, these results suggest that sex-specific phenotypic expression may play an important role in the interaction between adiposity and muscle deterioration. In this context, the elevated FMR observed in these phenotypes may reflect an increased ectopic lipid burden within skeletal muscle, which is consistent with processes of myosteatosis and reduced muscle quality [28]. At the cellular level, such alterations have been linked to mitochondrial dysfunction, which is considered a central mechanism in the development of insulin resistance and broader metabolic impairment [29,30,31]. 

Accordingly, the phenotypes identified in the present study may reflect not only differences in BC but also distinct metabolic states with potentially divergent trajectories of functional decline. The wide range of sex-specific differences in body composition observed in this study reflects a fundamental biological dimorphism driven by distinct hormonal, chromosomal, and metabolic mechanisms [32]. Estrogen predominantly directs adiposity toward a subcutaneous and gluteofemoral distribution while enhancing lipid oxidation [33], whereas testosterone maintains anabolic dominance over skeletal muscle accretion. These divergent physiological environments systematically result in higher fat mass ratios (FMR) in women and greater appendicular lean mass in men. Therefore, the sex-stratified analytical framework applied here is methodologically essential; performing pooled clustering would conflate biologically distinct populations, masking sex-specific physiological traits and yielding phenotypes of limited clinical interpretability.

With respect to the male “sarcopenia risk/lean” phenotype, the reduced ASMI may plausibly be associated with the long-term interaction of insufficient protein intake and inadequate mechanical loading, both of which may limit the optimal development and maintenance of skeletal muscle during adulthood. Over time, this may substantially increase vulnerability to frailty and adverse aging-related outcomes [34]. Some studies suggest that consuming protein and engaging in resistance training may serve as preventive strategies to promote muscle mass and function as we age [35,36]. This study analyzes a sample of 2710 individuals, divided into men and women, on the basis of data clustering and the validation of silhouette metrics. This approach enhanced the objectivity of the segmentation of the analyzed individuals while reducing the reliance on predefined clinical assessment criteria. Such models offer an opportunity to analyze biological structures that aim to go beyond conventional assessments, as concluded in other clinical studies that have employed clustering strategies [37,38]. It is important to acknowledge that this study did not collect data on dietary intake or habitual physical activity levels, which represent key modifiable determinants of body composition phenotype. The absence of these variables precludes behavioral attribution of the identified phenotypic profiles and constitutes a methodological limitation that should be considered when interpreting the results. Future research should incorporate validated tools for dietary assessment and objective physical activity monitoring (e.g., accelerometry) to evaluate their contribution to phenotypic classification and to inform targeted preventive interventions.

Furthermore, the results revealed that assessing the interactions between muscle and fat indicators provides a much broader and more consistent framework than analyzing each tissue separately. Previous studies have highlighted the need to consider both muscle mass and fat mass to better understand sarcopenia and sarcopenic obesity [39]. Similarly, the imbalance between different body tissues (muscle and adipose mass) could provide a pathophysiological profile driven by the negative interaction between adipose tissue and skeletal muscle [40]. The proposed mechanisms underlying this interaction include lipid toxicity within myocytes, chronic low-grade inflammation, and increased oxidative stress, all of which may contribute to impaired muscle function and tissue quality [41]. The current results support this perspective, particularly because the female “sarcopenic obesity” phenotype presented the highest FMR values, which is consistent with a marked imbalance between adipose and muscular compartments and suggests substantial deterioration in muscle quality.

The relevance of this ratio-based perspective is supported by Torres-Costoso et al. [42], who, after conducting cluster analyses in pediatric populations, noted that the fat-to-muscle ratio can provide more accurate information about the risk of sarcopenia than traditional indicators. They also reported that adiposity is not invariably associated with an increased risk of sarcopenia, particularly in individuals with relatively preserved lean mass and adequate muscular fitness, a phenomenon related to the ‘fat but fit’ paradox [43,44]. While the present study did not directly assess physical fitness, identifying phenotypes based on the fat–muscle ratio reinforces the importance of considering the dynamic balance between these compartments when evaluating body composition-related risk. Thus, the present results extend this conceptual framework to adulthood and suggest that the interaction between adiposity and muscle reserve provides a more comprehensive picture than isolated indicators of body size.

This study offers a methodological perspective aimed at providing insights derived from the use of ML techniques for BC assessment, with the goal of moving beyond stationary models to evaluate biological processes that do not follow a linear pattern. Urzi et al. [45], for example, applied predictive ML models in older adults via random forest approaches and achieved high discriminatory performance for sarcopenia detection, with an area under the curve of 0.95 and an accuracy of 93.62%, which was further improved through feature selection and SHAP-based interpretability techniques. 

These findings reinforce the potential value of ML in the identification of sarcopenia-related risk, although the requirement for multimodal data may limit its clinical applicability in some settings. In contrast, K-means clustering offers the advantage of exploring phenotypic heterogeneity without requiring prior outcome labeling. In the present study, this feature was particularly useful because it enabled the identification of biologically differentiated subgroups that may represent early stages in the pathway toward clinically detectable sarcopenia or sarcopenic obesity. Accordingly, both unsupervised and supervised ML approaches may have complementary value in future BC research and clinical screening.

### 4.1. Future Perspectives

Future research could explore different study designs, including longitudinal studies that allow for the assessment of phenotype changes across different population samples. It would also be valuable to align these phenotypic classifications with serum biomarkers, such as myostatin or inflammatory markers, together with objective physical performance tests to establish whether the clusters identified by the algorithm truly reflect distinct functional states and differential mortality risk. Finally, the present methodological framework could support the development of simplified clinical tools, such as mobile applications or web-based calculators, capable of classifying individuals into phenotypic clusters using a limited number of easily accessible anthropometric variables. Such an approach could facilitate precision-oriented screening in primary care and exercise science settings, particularly during earlier stages of adult life, and support multidisciplinary strategies aimed at reducing the latent risk associated with phenotypes such as “sarcopenia risk/lean” and “sarcopenic obesity”.

### 4.2. Limitations

Several limitations should be considered when interpreting the present findings. First, the cross-sectional design precludes any causal inference regarding the relationship between the identified phenotypes and subsequent physiological or clinical alterations, limiting interpretation to observational associations. Second, the absence of direct functional measures, such as muscle strength or physical performance tests, as well as the lack of biochemical markers, restricts the ability to confirm clinical sarcopenia according to current consensus-based diagnostic criteria. Therefore, the phenotypes identified in this study should be interpreted as risk profiles rather than definitive clinical diagnoses. Third, the relatively young adult age profile of the sample may limit the extrapolation of these results to older populations, in which the prevalence, expression, and clinical consequences of sarcopenia are likely to differ. Finally, although the unsupervised clustering approach was useful for identifying latent biological patterns, the incorporation of additional functional, behavioral, and metabolic variables would likely improve the robustness, interpretability, and clinical relevance of the proposed model.

## 5. Conclusions

We identified distinct adult BC phenotypes based on the interaction between muscle mass and adiposity via an unsupervised ML approach. The findings show that BMI alone is insufficient to detect clinically relevant risk profiles, particularly those characterized by unfavorable muscle–fat imbalance despite apparently normal body weight. The integration of the ASMI and FMR enabled the detection of sex-specific phenotypes associated with potentially increased sarcopenia-related and metabolic risk. In men, the “sarcopenia risk/lean” phenotype revealed reduced muscle reserve despite nonobese BMI values, whereas in women, the “sarcopenic obesity” phenotype was characterized by a marked imbalance between adiposity and muscle quality.

Overall, these results indicate that BC phenotyping based on muscle–fat relationships may provide a more informative framework than conventional anthropometric assessment for the early identification of adults at risk. This approach may support more targeted preventive strategies aimed at preserving muscle health, reducing excess adiposity, and improving metabolic outcomes.

## Figures and Tables

**Table 1:** Each of the different validation indicators was grouped according to sex.

Sex	*n*	Silhouette Score	Inertia	Calinski–Harabasz Index	Davies–Bouldin Index
Men	803	0.317	2007.6	399.9	1.174
Women	1907	0.307	4528.1	1052.7	1.099

**Table 2:** Anthropometric characteristics, BC indices, and tissue distribution profiles of the study population stratified by sex.

Variable	Total (*n* = 2710)	Women (*n* = 1907)	Men (*n* = 803)	*p* Value
Age (years)	37.72 ± 11.62	38.14 ± 11.73	36.72 ± 11.28	<0.001
Height (m)	1.64 ± 0.10	1.59 ± 0.06	1.76 ± 0.08	<0.001
Body weight (kg)	78.38 ± 15.83	75.46 ± 15.44	85.33 ± 14.55	<0.001
Fat mass (kg)	28.11 ± 12.19	30.90 ± 11.46	21.49 ± 11.28	<0.001
Muscle mass (kg)	28.08 ± 6.61	24.59 ± 3.61	36.36 ± 4.34	<0.001
Body fat (%)	35.19 ± 10.64	39.84 ± 7.53	24.16 ± 8.63	<0.001
Relative muscle mass (%)	36.11 ± 6.35	33.14 ± 4.13	43.18 ± 4.95	<0.001
BMI (kg/m^2^)	29.27 ± 5.91	29.90 ± 6.08	27.76 ± 5.19	<0.001
SMI (kg/m^2^)	10.32 ± 1.47	9.71 ± 1.16	11.76 ± 1.05	<0.001
ASMI (kg/m^2^)	7.57 ± 1.05	7.11 ± 0.82	8.65 ± 0.69	<0.001
FMR	1.06 ± 0.47	1.25 ± 0.38	0.59 ± 0.31	<0.001
Trunk-to-limb fat ratio	1.24 ± 0.16	1.18 ± 0.12	1.38 ± 0.15	<0.001

**ASMI**: Appendicular Skeletal Muscle Mass Index; **BMI**: Body Mass Index; **FMR**: Fat-to-Muscle Ratio; **SMI**: Skeletal Muscle Index.

**Table 3:** Male Body Composition phenotypes identified through clustering analysis (*n* = 803).

Variable	Athletic/Protector (*n* = 450)	Metabolic Obesity (*n* = 181)	Sarcopenia Risk/Lean (*n* = 172)	*p* Value
Age (years)	36.75 ± 9.43 (b)	39.51 ± 11.80 (a)	33.73 ± 14.12 (c)	<0.001
Height (m)	1.78 ± 0.09 (a)	1.73 ± 0.07 (b)	1.73 ± 0.08 (b)	<0.001
Body weight (kg)	81.43 ± 6.16 (b)	106.21 ± 13.24 (a)	73.55 ± 8.18 (c)	<0.001
BMI (kg/m^2^)	25.93 ± 2.41 (b)	35.37 ± 4.48 (a)	24.54 ± 3.12 (c)	<0.001
Fat mass (kg)	16.50 ± 4.69 (b)	38.02 ± 10.41 (a)	17.14 ± 7.13 (b)	<0.001
Muscle mass (kg)	37.03 ± 2.93 (b)	38.98 ± 5.02 (a)	31.84 ± 3.24 (c)	<0.001
Body fat (%)	20.13 ± 4.93 (c)	35.47 ± 6.46 (a)	22.78 ± 7.83 (b)	<0.001
Relative muscle mass (%)	45.55 ± 2.82 (a)	36.88 ± 3.86 (c)	43.59 ± 4.60 (b)	<0.001
SMI (kg/m^2^)	11.76 ± 0.64 (b)	12.91 ± 0.98 (a)	10.57 ± 0.57 (c)	<0.001
ASMI (kg/m^2^)	8.60 ± 0.36 (b)	9.49 ± 0.64 (a)	7.91 ± 0.39 (c)	<0.001
FMR	0.45 ± 0.14 (c)	0.99 ± 0.31 (a)	0.55 ± 0.25 (b)	<0.001
Trunk-to-limb fat ratio	1.46 ± 0.09 (a)	1.31 ± 0.14 (b)	1.25 ± 0.15 (c)	<0.001

**ASMI**: Appendicular Skeletal Muscle Mass Index; **BMI**: Body Mass Index; **FMR**: Fat-to-Muscle Ratio; **SMI**: Skeletal Muscle Index. Note: Data are presented as the means ± SDs. Different letters indicate significant post hoc differences according to Tukey’s HSD test (α = 0.05).

**Table 4:** Female Body Composition phenotypes identified through clustering analysis (n = 1907).

Variable	Compensatory Muscle (*n* = 573)	Normal/Lean (*n* = 684)	Sarcopenic Obesity (*n* = 650)	*p* Value
Age (years)	37.37 ± 9.73 (b)	36.04 ± 12.38 (b)	41.02 ± 12.09 (a)	<0.001
Height (m)	1.61 ± 0.05 (a)	1.58 ± 0.05 (b)	1.58 ± 0.06 (b)	<0.001
Body weight (kg)	73.09 ± 8.07 (b)	62.93 ± 7.46 (c)	90.73 ± 13.41 (a)	<0.001
BMI (kg/m^2^)	28.05 ± 2.83 (b)	25.24 ± 2.90 (c)	36.43 ± 4.91 (a)	<0.001
Fat mass (kg)	25.45 ± 5.72 (b)	23.38 ± 5.60 (c)	43.63 ± 8.70 (a)	<0.001
Muscle mass (kg)	26.50 ± 2.67 (a)	21.53 ± 2.01 (b)	26.13 ± 3.50 (a)	<0.001
Body fat (%)	34.51 ± 5.15 (c)	36.71 ± 5.50 (b)	47.84 ± 3.42 (a)	<0.001
Relative muscle mass (%)	36.41 ± 2.97 (a)	34.42 ± 3.01 (b)	28.90 ± 1.89 (c)	<0.001
SMI (kg/m^2^)	10.15 ± 0.69 (b)	8.62 ± 0.57 (c)	10.47 ± 1.08 (a)	<0.001
ASMI (kg/m^2^)	7.36 ± 0.48 (b)	6.33 ± 0.45 (c)	7.71 ± 0.71 (a)	<0.001
FMR	0.97 ± 0.21 (c)	1.09 ± 0.25 (b)	1.67 ± 0.23 (a)	<0.001
Trunk-to-limb fat ratio	1.28 ± 0.07 (a)	1.16 ± 0.09 (b)	1.11 ± 0.12 (c)	<0.001

**ASMI**: Appendicular Skeletal Muscle Mass Index; **BMI**: Body Mass Index; **FMR**: Fat-to-Muscle Ratio; **SMI**: Skeletal Muscle Index. Note: Values are presented as the means ± SDs. Different letters indicate significant post hoc differences according to Tukey’s HSD test (α = 0.05).

## Data Availability

The data presented in this study are available on request from the corresponding author.

## Appendix A

**Table A1:** Clustering Model Configuration (K-Means).

Parameter	Value
Algorithm	K-Means
Initialization	k-means++ (centroid optimization)
K (Clusters)	3 (fixed per sex)
n_init (Restarts)	20 (independent runs)
max_iter	500 (maximum iterations per run)
Distance Metric	Euclidean in multivariate space
Standardization	Z-Score (Standard Scaler) by sex
Random Seed	42 (for reproducibility)

**Table A2:** Model Quality and Validation Metrics.

Metric	Females (*n* = 1907)	Males (*n* = 803)
Silhouette Score	0.307	0.317
Bootstrap Silhouette (Mean ± SD)	0.308 ± 0.006	0.320 ± 0.010
95% Bootstrap CI	[0.297, 0.320]	[0.301, 0.341]
Calinski-Harabasz Index	1052.7	399.9
Davies-Bouldin Index	1.099	1.174
Inertia	4528.1	2007.6
